# Hsa-circ-0006091 modulates the proliferation of hepatocellular carcinoma via the miR-622/CCNB1 axis

**DOI:** 10.55730/1300-0144.5703

**Published:** 2023-05-31

**Authors:** Xiaofeng ZHAO, Ling HE

**Affiliations:** National Demonstration Center for Experimental Basic Medical Science Education, Xuzhou Medical University, Xuzhou City, China

**Keywords:** Hepatocellular carcinoma, circ-0006091, miR-622, cyclin B1, migration, invasion

## Abstract

**Background/aim:**

Hepatocellular carcinoma (HCC) is a common type of cancer. We hypothesize that circular RNA-0006091 (circ-0006091) affects the progression of HCC. The study aims to investigate the effect of circ-0006091 in HCC cells.

**Materials and methods:**

The levels of circ-0006091, microRNA-622 (miR-622), and cyclin B1 (CCNB1) were assayed using qRT-PCR and western blotting. The metastasis of the HCC cells was measured with wound healing and transwell assays. The protein expression levels of MMP-2 and MMP-9 were assayed with western blotting. Dual-luciferase reporter and RNA-pulldown assays were used to determine the link between miR-622 and circ-0006091 or CCNB1. Mice-based tests were used to determine the effect of circ-0006091 on the proliferation of HCC cells.

**Results:**

The levels of circ-0006091 and CCNB1 were increased in the HCC cells, but miR-622 was down-regulated. Deficiency of circ-0006091 reduced the metastasis of the HCC cells, and silencing of circ-0006091 decreased the activities of MMP-2 and MMP-9 in the same cells. Circ-0006091 modulated the CCNB1 level in the HCC cells via miR-622. Silencing of circ-0006091 suppressed the proliferation of the HCC cells in vivo.

**Conclusion:**

Circ-0006091 regulated HCC cell metastasis via the miR-622/CCNB1 axis, a possible therapeutic target in managing HCC.

## 1. Introduction

Hepatocellular carcinoma (HCC) is a common cancer and a leading cause of cancer-related death worldwide [1]. Currently, the primary management methods for HCC comprise surgery, chemotherapy, radiotherapy, and systemic therapy [2]. Nevertheless, due to the strong possibility of HCC cells metastasizing, the recurrence rate of HCC within 5 years after surgery is high (70%) [3]. Additionally, cirrhosis is one of the primary causes of HCC [4]. The curative effects of medicines commonly used to treat HCC are limited; sorafenib is an example of such a drug [5]. Hence, it is important to examine the molecular mechanism involved in HCC to expedite the development of management strategies for the disease.

Circular RNAs (circRNAs) have multiple biological functions and apparent tissue specificity [6–8]. Research has revealed that circRNAs have a central role in cancers. For instance, the content of circ-ITCH was decreased in HCC, and circ-ITCH participation modulated HCC cell growth [9]. Additionally, circ-CDYL is a crucial regulatory factor in advancing HCC [10]. A prior study showed that the content of cSMARCA5 was decreased in HCC tissues and that SMARCA5 inhibited HCC cell metastasis [11]. Moreover, hsa-circ-101280 contributed to the proliferation of HCC cell growth and inhibited cell apoptosis [12]. Downregulation of circ-0038718 repressed metastasis in HCC [13]. Zhang et al. [14] reported that the content of circ-0006091 was up-regulated in HCC, and circ-0006091 acted as a marker for HCC. However, the function of circ-0006091 in HCC cells and the identity of the modulating factors are unknown.

MicroRNAs (miRNAs) are regulatory factors that modulate various cell functions [15]. For example, miR-296 inhibited cell invasion and metastasis in HCC [16]. Additionally, miR-296-5p repressed the EMT-associated metastasis of HCC cells [17] and inhibited papillary thyroid carcinoma [18]. Furthermore, miR-211 facilitated autophagy-related apoptosis in cervical cancer cells [19], and miR-338-3p modulated the growth of cervical cancer cells [20]. Liu et al. reported that miR-622 might be a novel prognostic factor for HCC [21]. That study was carried out to determine the influence of miR-622 on HCC.

Cyclin B1 (CCNB1) regulates mitosis of the mammalian cells and plays a key role in its initiation [22]. The abundance of CCNB1 was boosted in pancreatic cancer (PC) tissues, and the knockdown of CCNB1 abrogated the proliferation of PC cells by activating the p53 signaling pathway [23]. Moreover, CCNB1 acted as a prognostic biomarker for HCC [24]. Xia et al. reported that CCNB1 modulated cell growth, metastasis, and sorafenib resistance in HCC [25]. Furthermore, the content of CCNB1 was increased in HCC [26], but the influence of CCNB1 in HCC remains unclear. The present study aims to investigate the effect of circ-0006091 on HCC and its underlying mechanism. Hopefully, tThe findings will be useful in identifying some potential therapeutic targets for HCC.

## 2. Materials and methods

### 2.1. Tissue collection

The samples were collected from Xuzhou Medical University. In all, 47 pairs of HCC and normal tissues were collected. Informed consent was obtained from all subjects before any research-related tests were initiated. The patient selection criteria are outlined in the following section.

#### 2.1.1. Inclusion criteria

Patients in the following categories were included: those with a confirmed HCC diagnosis; patients over the age of 18; patients opting for voluntary participation in the study who signed the informed consent form; those with no prior exposure to chemotherapy, radiotherapy, or surgical treatment; and patients with no comorbidities, such as cardiovascular disease and immune system disorders. Patients who did not use drugs or have prior treatment affecting circRNA expression were also included.

#### 2.1.2. Exclusion criteria

Patients unable to provide sufficient information, those who refused to participate in the study, who had other cancers or serious illnesses, who had prior exposure to chemotherapy, radiotherapy, or surgical treatment, and who used drugs or treatments that may affect circRNA expression were excluded. In addition, pregnant or lactating patients and those with psychiatric disorders or cognitive impairments were not included in the study.

All protocols used in the study were approved by the Ethics Committee of Xuzhou Medical University (approval No. 202010A050, 08/05/2019). The samples were snap-frozen and stored at −80 °C.

### 2.2. Cell lines and transfection

HCC cell lines (HCCLM3, Huh-7, and MHCC97L) and the normal adult liver epithelial cell line (THLE-2) were obtained from the JCRB cell bank (Osaka, Japan). The cells used in this research were maintained in DMEM (Solarbio, Beijing, China) with 10% FBS (Solarbio), penicillin (100μg/mL; Solarbio), and streptomycin (100 U/mL; Solarbio). The cells were cultured at 37°C in a 5% CO_2_ incubator. The siRNA targeting circ-0006091 (si-circ-0006091#1 and si-circ-0006091#2) and control (si-NC), shRNA targeting circ-0006091 (sh-circ-0006091) and control (sh-NC), miR-622 mimic and inhibitor (miR-622 and anti-miR-622) and controls (miR-NC and anti-NC), as well as siRNA targeting CCNB1 (si-CCNB1) and control (si-NC), were obtained from GenePharma (Shanghai, China). Cell transfection was implemented using Lipofectamine 2000 (Invitrogen, Carlsbad, CA, USA).

### 2.3. qRT-PCR and Actinomycin D (Act D) assay

Total RNA was isolated using TRIzol (TaKaRa, Tokyo, Japan). A Prime Script RT kit (TaKaRa) or a HiScript II 1st Strand cDNA Synthesis Kit (TaKaRa) was utilized to reverse transcribe the total RNA to cDNA. Next, qRT-PCR was carried out using a SYBR Green Master Mix (Solarbio) with GAPDH and U6 as internal references. The relative gene expression level was calculated using the 2^−ΔΔCT^ method. The sequences of primers used are presented in the Table. For the Act D assay, HCC cells were incubated with Act D (2 mg/mL; Solarbio) for 6, 12, and 24 h. The mRNA expression levels of circ-0006091 and RGS12 were assayed using qRT-PCR.

### 2.4. Wound-healing assay

HCC cells subjected to different treatments were seeded in 6- well plates and cultured until an 80% confluence was reached. A wound was created on the HCC cell layer using a 200-μL pipette tip. The migration of individual cells was evaluated under a microscope (Olympus, Tokyo, Japan) at 0 and 24h.

### 2.5. Transwell assay

HCC cells with different transfections were seeded in the upper chamber of the transwell (Solarbio) with serum-free DMEM (Solarbio). The lower chamber contained DMEM (Solarbio) with 10% FBS (Solarbio). The migrated cells were treated with 4% paraformaldehyde (Solarbio) and stained with 0.5% crystal violet (Invitrogen). After 24 h, the migrated cells were examined under a microscope (Olympus), and the percentage of cell migration was calculated. To evaluate cell invasion, the upper chamber of the transwell was coated with Matrigel (Solarbio) beforehand, and the other steps were carried out in line with the migration assay.

### 2.6. Western blot assay

Total protein was extracted from the cell samples by lysing with RIPA buffer (Invitrogen), and the proteins were resolved on 10% SDS-PAGE, followed by electrotransfer PVDF membranes (Invitrogen). After blocking using fat-free milk (0.5%), the membranes were incubated overnight at 4°C with primary antibodies (i.e. anti-CCNB1 (ab32053; 1:1,000; Abcam, Cambridge, MA, USA), anti-MMP2 (ab92536; 1:1000; Abcam), anti-MMP9 (ab76003; 1:200; Abcam), and anti-β-actin (ab8226; 1:1000; Abcam)). The membranes were then incubated with horseradish peroxidase-conjugated secondary antibody (ab205718; 1:2500; Abcam) for 1 h at room temperature. The bands were analyzed using ImageJ software.

### 2.7. Dual-luciferase reporter assay

The circ-0006091 or CCNB1 3’ UTR fragments with the WT or MUT miR-622 binding sites were placed in the pMIR reporter vectors (Promega, Madison, WI, USA). Subsequently, the HCCLM3 and Huh-7 cells were transfected with the vectors mentioned above and the miR-622 mimic or miR-NC. After 48 h, a Dual-Luciferase Reporter Assay System (Promega) was used to assay luciferase activity.

### 2.8. RNA pulldown assay

A circ-0006091 probe, a control probe, and a miR-622 probe (bio-miR-622) and bio-NC were obtained from GenePharma. The probes were coated with C-1 magnetic beads (Invitrogen). The HCC cells were treated with lysis solution (Invitrogen) and the previously -mentioned probes overnight. Finally, the precipitates were isolated and purified using an RNeasy Mini Kit (Invitrogen). The level of circ-0006091 in the RNA complexes was determined using qRT-PCR.

### 2.9. Animal test

The mouse experiment was carried out with the approval of the animal ethics committee of Xuzhou Medical University (No. 202010A050). A total of 12 male BALB/c nude mice aged 4 weeks were obtained from the Shanghai Laboratory Animal Company (SLAC, Shanghai, China). Huh7 cells (1.5 × 10^6^) with sh-circ-0006091 or sh-NC were injected into the mice subcutaneously. Tumor volume was calculated every 10 days using the following formula:


(2) $$Tumor\;volume=(length\times {width}^{2})$$

The mice were euthanized after 30 days, and the tumor tissues were used in additional tests.

### 2.10. Statistical analysis

Data are presented as mean ± SD. Group comparison was initiated using ANOVA or Student’s t-test. Statistical analysis was conducted with SPSS, and p < 0.05 indicated a statistically significant difference.

## 3. Results

### 3.1. circ-0006091 was highly expressed in HCC

**Figure 1:**
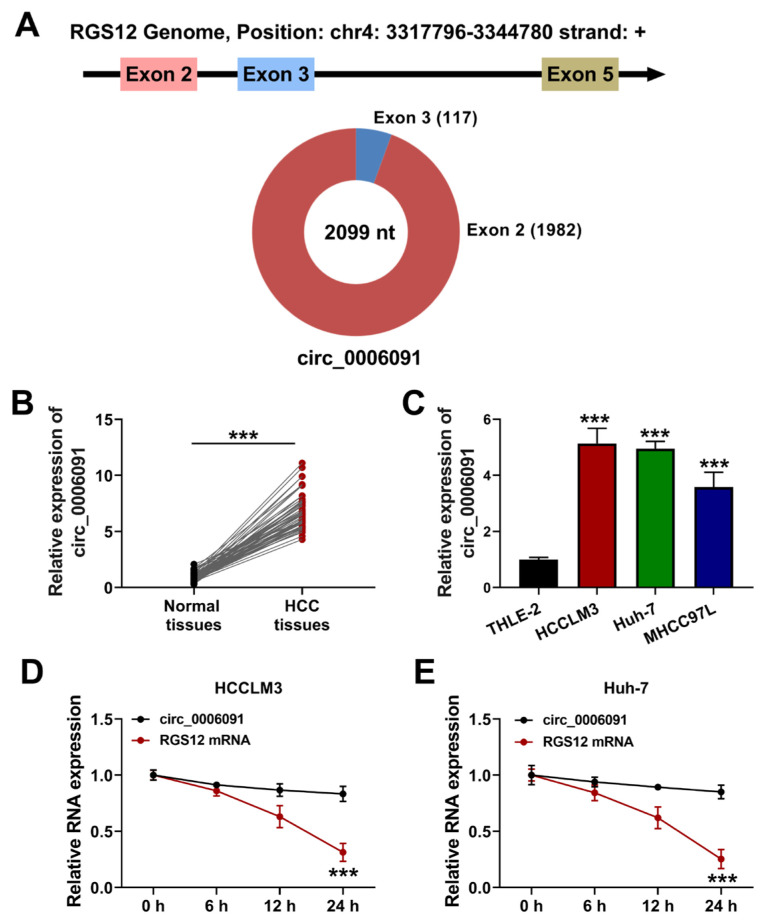
Circ-0006091 level heightened in HCC. (a) Schematic illustration of circ-0006091 formation; (b) Expression of circ-0006091 in HCC tissues and normal tissues was detected by qRT-PCR; (c) Expression of circ-0006091 in HCC cells (HCCLM3, Huh-7, and MHCC97L) and THLE-2 cells assessed by qRT-PCR; (d and e) Circ-0006091 and RGS12 relative mRNA levels in HCCLM3 and Huh-7 cells were quantified by qRT-PCR. ***p < 0.001.

As shown in Figure 1a, circ-0006091 is located on chromosome 4 (chr4: 3317796-3344780) at a length of 2099 nucleotides and was derived from the RGS12 host gene. The expression of circ-0006091 was significantly upregulated in HCC tumor tissues relative to normal tissues (Figure 1b; p < 0.001). The expression of circ-0006091 was also upregulated in the HCCLM3, Huh-7, and MHCC97L cells, compared to the THLE-2 cells (Figure 1c; p < 0.001). However, the upregulation of circ-0006091 was more evident in the HCCLM3 and Huh-7 cells. Therefore, these two types of cells were selected for the subsequent experiments. Moreover, the Act D assay showed that circ-0006091 was resistant to Act D treatment, while RGS12 mRNA was not (Figures 1d and 1e; p < 0.001). These findings suggest that circ-0006091 was upregulated and had a circular structure in HCC.

### 3.2. Deficiency of circ_0006091 repressed metastasis of the HCC cells

**Figure 2:**
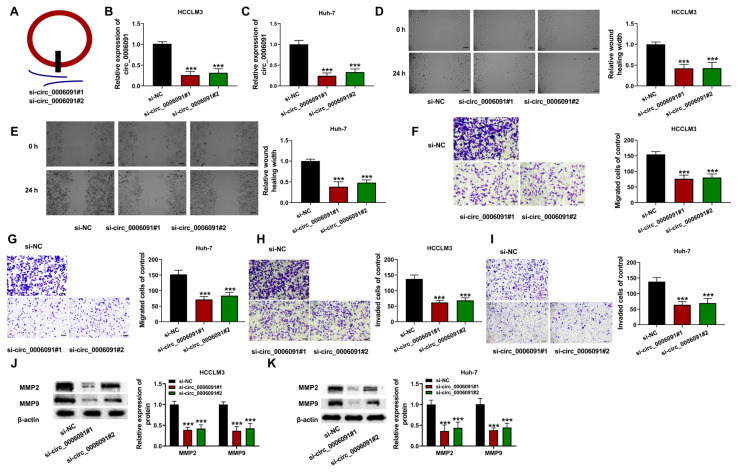
Effect of circ-0006091 knockdown on HCC cells. The HCCLM3 and Huh-7 cells were transfected with si-NC or si-circ-0006091. Each cell index was determined 48 h after transfection. (a) Schematic illustration of si-circ-0006091#1 and si-circ-0006091#2; (b and c) Expression of circ-0006091 evaluated with qRT-PCR; (d and e) Cell migration determined using wound healing assay (scale bar = 100μm). (f–i) Cell migration and invasion estimated using a transwell assay (scale bar = 50μm); (j and k) Relative protein expression levels of MMP-2 and MMP-9, as assayed using western blotting. ***p < 0.001.

The influence of circ-0006091 on the HCC cells was then investigated. Figure 2a shows the schematic diagram of two siRNAs of circ-0006091. The expression of circ-0006091 in the HCC cells was decreased by transfection of si-circ-0006091 (si-circ-0006091#1 and si-circ-0006091#2; Figures 2b and 2c; p < 0.001). In addition, silencing of circ-0006091 decreased the migration (Figures 2d–2g; p < 0.001) and invasion (Figures 2h and 2i; p < 0.001) of the HCC cells. Moreover, the expressions of metastasis-associated proteins MMP-2 and MMP-9 were down-regulated by knockdown of circ-0006091 (Figures 2j and 2k; p < 0.001). These results demonstrate that deletion of circ-0006091 led to reduced metastasis of the HCC cells.

### 3.3. Circ-0006091 targeted miR-622

**Figure 3:**
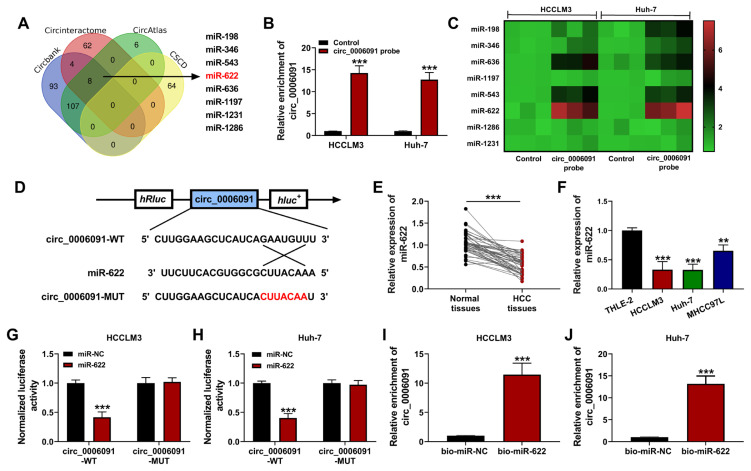
MiR-622 was a target of circ-0006091. (a) Venn diagram showing the predicted miRNAs of Circbank, Circinteractome, CircAtlas, and CSCD; (b) Relative enrichment of circ-0006091 evaluated using qRT-PCR; (c) Heat map of the relative enrichment of the predicted miRNAs in HCCLM3 and Huh-7 cells; (d) Targeted sites of miR-622 and circ-0006091; (e) Expressions of miR-622 in HCC tissues and normal tissues determined using qRT-PCR; (f) Expression of miR-622 in HCC cells (HCCLM3, Huh-7, and MHCC97L) and THLE-2 cells assayed using qRT-PCR; (g and h) Luciferase activities of HCCLM3 and Huh-7 cells co-transfected with miR-622 mimics or miR-NC mimics and luciferase reporters containing circ-0006091-WT or circ-0006091-MUT transcript; (i and j) Association between circ-0006091 and miR-622 revealed using RNA-pulldown. **p < 0.01, ***p < 0.001.

Circbank (http://www.circbank.cn/), Circinteractome (https://circinteractome.nia.nih.gov), CircAtlas (http://circatlas.biols.ac.cn/), and CSCD (http://www.webofknowledge.com/) were used to predict the target of circ-0006091. As shown in Figure 3a, 8 miRNAs (i.e miR-198, miR-346, miR-543, miR-622, miR-636, miR-1197, miR-1231, and miR-1286) were identified as possible targets of circ-0006091.

The expression of circ-0006091 was enhanced in the HCC cells by treating it with a circ-0006091 probe (Figure 3B, p < 0.001). Heat map analysis showed that the expression of miR-622 was abridged in HCC, compared to control tissues (Figure 3c). Therefore, miR-622 was chosen for further experiments. Figure 3d shows the possible binding sites of miR-622 and circ-0006091. The expression of miR-622 was decreased in the HCC cells relative to the normal tissues (Figure 3e; p < 0.001). Furthermore, the miR-622 level was reduced in the HCCLM3, Huh-7, and MHCC97L cells, in contrast to the THLE-2 cells (Figure 3f; p < 0.001). The results of dual-luciferase reporter assay (Figures 3g and 3h; p < 0.001) and RNA knockdown (Figures 3i and 3j; p < 0.001) revealed that miR-622 targeted circ-0006091. Overall, the results suggest that circ-0006091 acted as a sponge for miR-622.

### 3.4. Circ-0006091 affected metastasis of the HCC cells through regulation of miR-622

**Figure 4:**
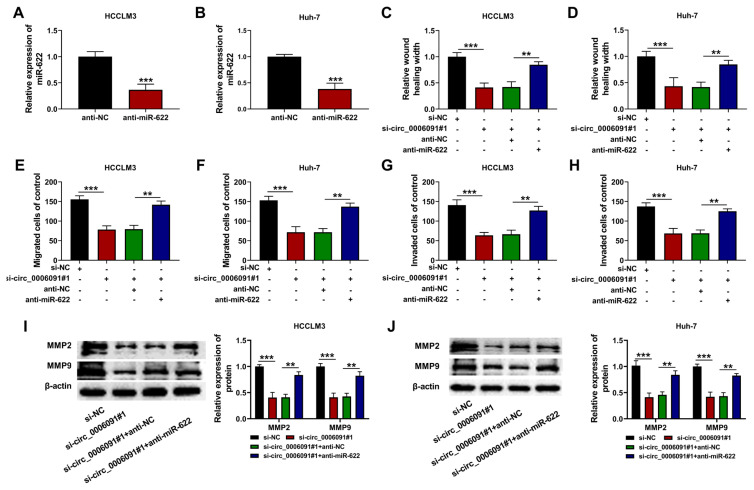
Circ-0006091 regulated metastasis in HCC cells via miR-622 HCCLM3, and Huh-7 cells were transfected with si-NC/si-circ-0006091#1/si-circ-0006091#1 + anti-NC/si-circ-0006091#1 + miR-622 mimics. Each cell index was determined 48 h after transfection. (a and b) Expressions of miR-622 in HCCLM3 and Huh-7 cells evaluated using qRT-PCR; (c and d) Cell migration measured using wound healing assay; (e–h) Cell migration and invasion estimated using transwell assay; (i and j) Relative protein levels of MMP-2 and MMP-9 determined using western blotting. **p < 0.01; ***p < 0.001.

As shown in Figure 4, anti-miR-622 transfection decreased the content of miR-622 in the HCC cells (Figures 4a and 4b; p < 0.001). In addition, silencing of circ-0006091 suppressed the migration (Figures 4c–4f; p < 0.001) and invasion (Figures 4g and 4h; p < 0.001) of the HCC cells, while miR-622 knockdown reversed these effects. Moreover, the down-regulation of miR-622 mitigated the effect of si-circ-0006091 on the activities of MMP-2 and MMP-9 (Figures 4i and 4j; p < 0.001). Thus, the deletion of circ-0006091 blocked the metastasis of the HCC cells through modulation of miR-622.

### 3.5. CCNB1 was bound to miR-622

**Figure 5:**
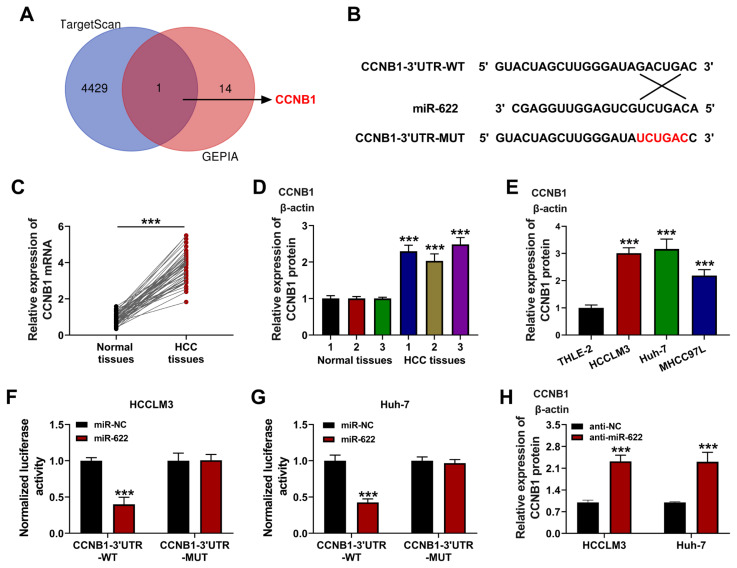
CCNB1 bound to miR-622. (a) Venn diagram showing the predicted genes of TargetScan and GEPIA; (b) Targeted sites of miR-622 and CCNB1; (c and d) Expression of CCNB1 in HCC tissues and normal tissues determined using qRT-PCR and western blot; (e) Expression of CCNB1 in HCC cells (HCCLM3, Huh-7, and MHCC97L) and THLE-2 measured using western blotting; (f and g) Luciferase activities of HCCLM3 and Huh-7 cells co-transfected with miR-622 mimics or miR-NC mimics and luciferase reporters containing CCNB1-3’UTR-WT or 9 CCNB1-3’UTR-MUT transcript assayed using dual-luciferase reporter assays; (h) Relative protein level of CCNB1. ***p < 0.001.

TargetScan (http://www.targetscan.org/) and GEPIA (http://gepia.cancer-pku.cn/) were used to predict the target of miR-622. As shown in Figure 5a, CCNB1 was identified as a possible target of miR-622. Figure 5b shows the ten potential binding sites of miR-622 and CCNB1. The content of CCNB1 was boosted in the HCC tissues compared to the normal tissues (Figures 5c and 5d; p < 0.001). Additionally, the CCNB1 level was higher in the HCCLM3, Huh-7, and MHCC97L cells than in the THLE-2 cells (Figure 5e; p < 0.001). Dual-luciferase reporter assay revealed that CCNB1 targeted miR-622 (Figures 5f and 5g; p < 0.001). Moreover, the CCNB1 content was boosted by downregulation of miR-622 (Figure 5h; p < 0.001). These results suggest that CCNB1 was bound to miR-622.

### 3.6. MiR-622 affected the metastasis of the HCC cells through modulation of CCNB1

**Figure 6:**
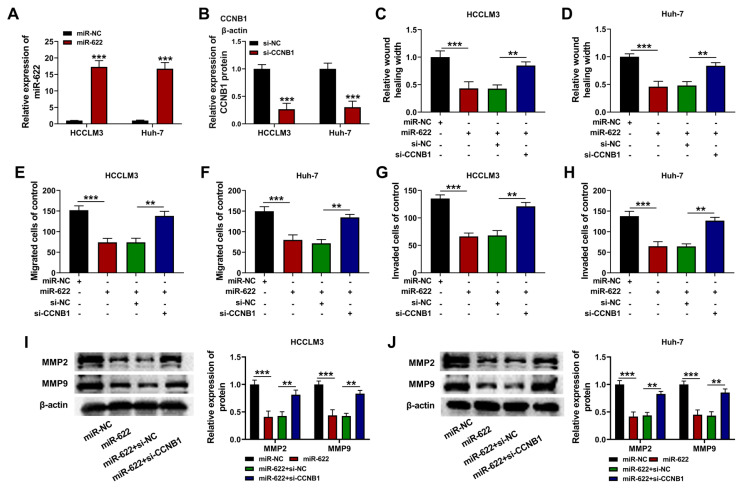
MiR-622 affected the metastasis of HCC cells through the regulation of CCNB1. HCCLM3 and Huh-7 cells were transfected with miR-NC/miR-622/miR-622 + si-NC/miR-622 + si-CCNB1. Each cell index was determined 48 h after transfection. (a) Expression of miR-622 evaluated with qRT-PCR. (B) Expression of CCNB1 assayed using western blotting; (c and d) Cell migration potential measured using wound healing assay; (e–h) Cell migration and invasion determined using transwell assay; (i and j) Levels of MMP-2 and MMP-9 assayed with western blot. **p < 0.01; ***p < 0.001.

**Figure 7:**
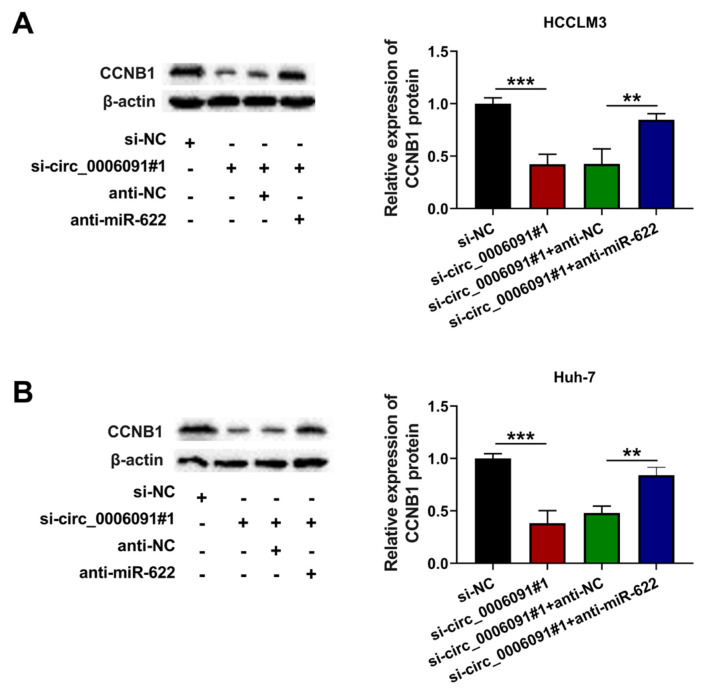
Circ-0006091 and miR-622 regulated the level of CCNB1. HCCLM3 and Huh-7 cells were transfected with si-NC/si-circ-0006091#1/si-circ-0006091#1 + anti-NC/si-circ-0006091#1 + miR-622 mimics. (a and b) Relative protein levels of CCNB1 quantified with western blot. **p < 0.01; ***p < 0.001.

Transfection of miR-622 mimic boosted the miR-622 content of the HCC cells (Figure 6a; p < 0.001). In addition, the content of CCNB1 in the HCC cells was decreased by transfection with si-CCNB1 (Figure 6b; p < 0.001), while miR-622 upregulation markedly reduced the migration (Figures 6c–6f; p < 0.001) and invasion (Figures 6g and 6h; p < 0.001) of the HCC cells. However, silencing of CCNB1 counteracted these effects. Furthermore, silencing CCNB1 mitigated the effect of miR-622 upregulation on the activities of MMP-2 and MMP-9 (Figures 6i and 6j; p < 0.001). The level of CCNB1 in the HCC cells was decreased by silencing circ-0006091, whereas it was enhanced by miR-622 downregulation (Figures 7a and 7b; p < 0.001). Silencing circ-0006091 abridged the metastasis of the HCC cells by modulating the miR-622/CCNB1 axis.

### 3.7. Silencing of circ-0006091 repressed the invasion of HCC in vivo

**Figure 8:**
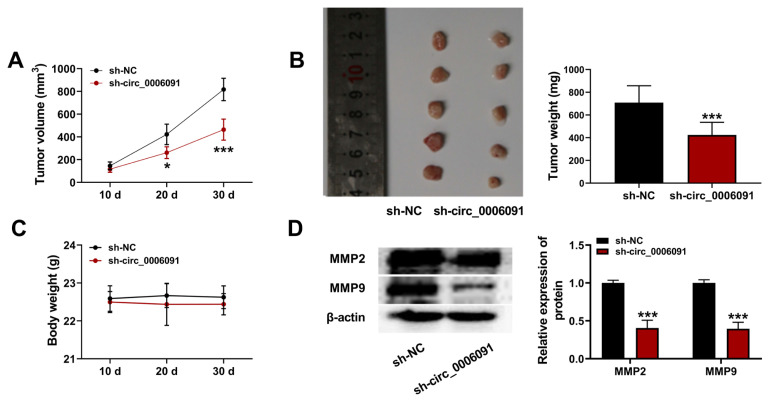
Lack of circ-0006091 inhibited the invasion potential of HCC cells. (a and b) Changes in tumor volume and weight; (c) Changes in the body weight of mice; (d) Relative protein levels of MMP-2 and MMP-9 quantified using western blotting. ***p < 0.001.

To investigate the effect of circ-0006091 in the progression of HCC in vivo, a xenograft tumor mouse model was established, as shown in Figure 8. The results show that silencing of circ-0006091 inhibited HCC growth (Figures 8a–8c; p < 0.001). In addition, the activities of MMP-2 and MMP-9 were inhibited in the sh-circ-0006091 group (Figure 8d; p < 0.001). These data confirm that a deficiency of circ-0006091 suppressed the proliferation and metastasis of HCC in mice.

## 4. Discussion

The incidence of HCC, which is higher in males than females, may be associated with certain lifestyle habits (e.g., alcoholism) [27,28]. Unfortunately, most individuals suffering from HCC are diagnosed in advanced stages of the disease and cannot benefit from the effective treatment period [29,30]. It is therefore essential to identify the regulatory targets of HCC. This study found that the content of circ-0006091 was up-regulated in HCC, similar to Zhang et al.’s study [14]. Many researchers have shown that circRNAs play an important role in HCC. For example, hsa-circ-0004018 has been implicated in HCC’s pathogenesis [31]. The circ-KIAA1429 level is increased in HCC, and circ-KIAA1429 upregulation accelerates the metastasis of HCC cells [32]. Moreover, it has been reported that circ-MMP-2 enhanced HCC cell metastasis by increasing MMP-2 content [33]. Furthermore, the content of circ-0000517 was boosted in HCC, and silencing of circ-0000517 repressed cell growth, metastasis, and glycolysis [34]. In another study, circ-0051443 arrested the cell cycle but expedited cell apoptosis in HCC [35]. In the present study, the downregulation of circ-0006091 reduced the metastasis of the HCC cells. In addition, silencing of circ-0006091 decreased the activities of MMP-2 and MMP-9 in the same cells. In a study by Ni et al. [36], the authors showed that downregulation of circ-0011385 suppressed the growth of HCC cells by modulating the miR-361-3p/STC2 axis. Furthermore, Huang et al. [37] reported that circ-104348 facilitated HCC invasion by targeting the miR-187-3p/RTKN2 axis. In the current study, it was found that circ-0006091 accelerated HCC cell metastasis via miR-622. This is consistent with findings from Ni et al. [36] and Huang et al. [37].

Targhazeh et al. demonstrated that the content of miR-622 was downregulated in prostate cancer (PCa) cells but that miR-622 enhanced apoptosis and reduced metastasis in PCa cells [38]. Orlandella et al. reported low levels of miR-622 in breast cancer tissues, determining that miR-622 affected the metastasis of breast cancer cells [39]. Fang et al. showed that upregulation of miR-622 decreased the metastasis and angiogenesis of colorectal cancer cells in vitro [40]. Yang et al. reported reduced levels of miR-622 in gastric cancer and found that miR-622 modulated gastric cancer cell growth and migration [41]. In the present study, the miR-622 level was decreased in HCC, similar to Song et al.’s study [42]. Additionally, it was found that miR-622 knockdown abolished the effect of downregulation of circ-0006091 on HCC cell metastasis. Circ-0006091 accelerated the metastasis of the HCC cells by regulating miR-622. Moreover, CCNB1 targeted miR-622.

Zhou et al. reported that CCNB1 content was accentuated in HCC and that CCNB1 might be a prognostic biomarker of HCC [43]. The present study found that CCNB1 content was augmented in HCC, similar to Zhou et al.’s findings [43]. Rong et al. indicated that CCNB1 modulated the HCC cell cycle through the regulation of DNA replication [44]. In the current study, it was also found that miR-622 regulated CCNB1 to affect the metastasis of the HCC cells. Circ-0006091 modulated the CCNB1 level by targeting miR-622.

Overall, these findings suggest that the levels of circ-0006091 and CCNB1 are increased, while the miR-622 level is decreased in HCC. For the first time, this study demonstrates that circ-0006091 facilitated the metastasis of HCC cells by modulating the miR-622/CCNB1 axis. This finding unravels HCC’s molecular basis and introduces potential therapeutic targets for the disease.

## Figures and Tables

**Table:** Sequences of the primers used for qRT-PCR.

Name		Primers for PCR (5’–3’)
hsa-circ-0006091	Forward	GGGAACTGGACTCACTCAGC
	Reverse	AGCACGTCTCATTGTTCCCT
RGS12	Forward	CTGCCGAGGCGACCG
	Reverse	TCCAAGACCAAGAGCACGTC
CCNB1	Forward	GCACTTCCTTCGGAGAGCAT
	Reverse	TGTTCTTGACAGTCCATTCACCA
miR-622	Forward	GCCGAGAAACATTCGCGGTGCA
	Reverse	CTCAACTGGTGTCGTGGAG
miR-198	Forward	GCCGAGGGTCCAGAGGGGAG
	Reverse	CTCAACTGGTGTCGTGGAG
miR-346	Forward	GCCGAGTGTCTGCCCGCATGC
	Reverse	CTCAACTGGTGTCGTGGAG
miR-543	Forward	GCCGAGAAACATTCGCGGTGCA
	Reverse	CTCAACTGGTGTCGTGGAG
miR-636	Forward	GCCGAGTGTGCTTGCTCGTCCC
	Reverse	CTCAACTGGTGTCGTGGAG
miR-1197	Forward	GCCGAGTAGGACACATGGTCTA
	Reverse	CTCAACTGGTGTCGTGGAG
miR-1231	Forward	GCCGAGGTGTCTGGGCGGAC
	Reverse	CTCAACTGGTGTCGTGGAG
miR-1286	Forward	GCCGAGTGCAGGACCAAGATG
	Reverse	CTCAACTGGTGTCGTGGAG
GAPDH	Forward	TACGGGTCTAGGGATGCTGG
	Reverse	TTTTGTCTACGGGACGAGGC
U6	Forward	CTCGCTTCGGCAGCACATATACT
	Reverse	ACGCTTCACGAATTTGCGTGTC
